# Pangenome Analysis of the Soilborne Fungal Phytopathogen *Rhizoctonia solani* and Development of a Comprehensive Web Resource: RsolaniDB

**DOI:** 10.3389/fmicb.2022.839524

**Published:** 2022-03-25

**Authors:** Abhinav Kaushik, Daniel P. Roberts, Abhinay Ramaprasad, Sara Mfarrej, Mridul Nair, Dilip K. Lakshman, Arnab Pain

**Affiliations:** ^1^Biological and Environmental Science and Engineering Division, King Abdullah University of Science and Technology (KAUST), Thuwal, Saudi Arabia; ^2^Sustainable Agricultural Systems Laboratory, United States Department of Agriculture-Agricultural Research Service (USDA-ARS), Beltsville, MD, United States; ^3^International Institute for Zoonosis Control, Hokkaido University, Sapporo, Japan

**Keywords:** basidiomycetous fungi, soilborne plant pathogen, *Rhizoctonia solani*, NGS—next generation sequencing, genomics, pangenome analyses, pathogenicity genes, genome database

## Abstract

Rhizoctonia solani is a collective group of genetically and pathologically diverse basidiomycetous fungi that damage economically important crops. Its isolates are classified into 13 Anastomosis Groups (AGs) and subgroups having distinctive morphology and host ranges. The genetic factors driving the unique features of *R. solani* pathology are not well characterized due to the limited availability of its annotated genomes. Therefore, we performed genome sequencing, assembly, annotation and functional analysis of 12 *R. solani* isolates covering 7 AGs and select subgroups (AG1-IA; AG1-IB; AG1-IC; AG2-2IIIB; AG3-PT, isolates Rhs 1AP and the hypovirulent Rhs1A1; AG3-TB; AG4-HG-I, isolates Rs23 and R118-11; AG5; AG6; and AG8), in which six genomes are reported for the first time. Using a pangenome comparative analysis of 12 *R. solani* isolates and 15 other Basidiomycetes, we defined the unique and shared secretomes, CAZymes, and effectors across the AGs. We have also elucidated the *R. solani*-derived factors potentially involved in determining AG-specific host preference, and the attributes distinguishing them from other Basidiomycetes. Finally, we present the largest repertoire of *R. solani* genomes and their annotated components as a comprehensive database, viz. RsolaniDB, with tools for large-scale data mining, functional enrichment and sequence analysis not available with other state-of-the-art platforms.

## Introduction

*Rhizoctonia solani* Kühn [teleomorph: *Thanatephorus cucumeris* (Frank) Donk] is considered one of the most destructive soil-borne plant pathogens causing various diseases including pre- and post-emergence damping-off of seedlings, crown and root rots, black scurf of potato, take-all of wheat, sheath blight of rice and maize, brown patch of turf, and postharvest fruit rots (Yang and Li, 2012; Amaradasa et al., 2013). This necrotrophic fungus infects a wide range of economically important plant species, belonging to more than 32 plant families and 188 genera, and is responsible for 15–50% of agricultural damage annually (Raaijmakers et al., 2009). Broadly, it is classified among 13 Anastomosis Groups (AGs) with distinctive morphology, physiology, pathogenicity host range, and highly divergent genetic composition (Gónzalez et al., 2016). Most *R. solani* AGs are further divided into subgroups which differ in pathogenicity, virulence, ability to form sclerotia, growth rate, and host range preference (Keijer et al., 1997). Although field isolates of *Rhizoctonia* infected plants are usually found to be infested with one or more AGs, each AG subgroup can still have its own host preference. For instance, *Arabidopsis thaliana* was found to be susceptible to AG2-1 sub-group isolates but resistant to AG8 isolates (Foley et al., 2013), which suggests that genetic divergence is the inherent characteristic of *Rhizoctonia* species.

Over the last two decades, our understanding of the genetic divergence among different *R. solani* AGs has improved to the point that it is now evident that all AGs and their sub-groups are genetically isolated, non-interbreeding populations (Gonzalez et al., 2001). The rapid and relatively low-cost of generation of genomic sequences and other “*omics*” datasets has played a significant role in furthering our understanding of the host-pathogen interactions and ecology of *Rhizoctonia* species. (Hane et al., 2014; Hossain et al., 2014; Anderson et al., 2016; Lakshman et al., 2016; Copley et al., 2017). The analysis of these genomic sequences and functional components revealed several novel or previously unrecognized classes of *R. solani* genes among different AGs that are involved in pathogenesis in a host-specific manner, e.g., effector proteins and carbohydrate-active enzymes (CAZymes) (Wibberg et al., 2016b). Additionally, analysis of differentially expressed genes in different isolates has enabled researchers to predict the adaptive behavior of this fungus in different hosts and the associated virulence (Zhang et al., 2017; Shu et al., 2019). However, the majority of this information has come from the analysis of isolates belonging to only a small number of AGs for which complete genome and/or transcriptome sequences are available. In fact, until now, draft genome assemblies belonging to only 4 of the 13 AGs have been reported viz. AG1-IA (Nadarajah et al., 2017; Ghosh et al., 2019; Lee et al., 2021), AG1-IB (Wibberg et al., 2015a,b), AG2-2IIIB (Wibberg et al., 2016b), AG3-Rhs1AP (Cubeta et al., 2014), AG3-PT isolate Ben-3 (Wibberg et al., 2017) and AG8 (Hane et al., 2014). This limited availability of genome sequences and the predicted proteomes across the 13 different AGs and their subgroups is one of the important barriers hindering the understanding of functional complexity and temporal dynamics in *R. solani* AGs and their subgroups.

In this study, we report whole-genome sequencing, assembly and annotation of 12 *Rhizoctonia* isolates from 7 AGs; of which genome sequences of three AGs (AG4, AG5, and AG6), two subgroups (AG1-IC and AG3-TB {or AG3-T5}) and a hypovirulent isolate (AG3-1A1) of the subgroup AG3-PT are being reported for the first time. The draft genome of the AG3-PT isolate 1AP (alternatively named as Rhs1AP) was previously reported (Cubeta et al., 2014), but was re-sequenced for comparative purposes, as AG3-1AP. To make these high-quality draft *R. solani* genomes and features readily accessible to a broad audience of researchers, we built a comprehensive and dedicated web resource, viz. RsolaniDB, for hosting and analyzing the available genomic information predicted at the transcript-, and protein-level in different *R. solani* AGs.

## Materials and Methods

### Isolation of Genomic DNAs for Sequencing

Details regarding *R. solani* isolates used for sequence analyses are presented in Supplementary Tables 1, 2. Fungal cultures were purified by the hyphal tip excision method (Bills et al., 1993) and maintained by sub-culturing on potato dextrose agar (PDA; Sigma Aldrich catalog # P2182, St. Louis, MO, United States). The PDA was amended with kanamycin (25 μg/ml) and streptomycin (50 μg/ml) to inhibit bacterial growth. Isolates were grown in potato dextrose broth (PDB; Sigma Aldrich catalog # P6685) at 100 rpm and 25°C for 4–6 days, mycelia collected by filtration through 2 layers of sterile cheese cloth, washed 2 X with sterile distilled water, gently squeezed and placed on 4 layers of paper towels to remove surface water, and then snap-frozen in liquid nitrogen and stored at –80°C till use. Genomic DNA was extracted from mycelia using both the CTAB method (Carlson et al., 1991) and a protocol recommended by the manufacturer (User-Developed Protocol: Isolation of genomic DNA from plants and filamentous fungi using the QIAGEN^®^ Genomic-tip, Qiagen, Inc., Germantown, MD, United States). RNA was extracted from fungal isolates and from tobacco detached leaves infected with corresponding fungal isolates, using the Qiagen RNeasy Plant Mini Kit (Qiagen, Inc.). Extracted genomic DNA and RNA was quantified with a Qubit Flex Fluorometer (Thermo Fisher Scientific, Waltham, MA, United States). AG and subgroup identity of the fungal isolates was verified by ITS-PCR, sequencing and homology analysis with nucleotide sequences available in the NCBI database (Sayers et al., 2020).

### RNA Extraction

*Nicotiana tabacum* seedlings were raised to the four-leaf stage on potting mix (Pro-mix, Premier Horticulture, Quakertown, PA, United States) in the greenhouse at ambient temperature (22°–24°C) and 4 h supplemental light with a mercury lamp. Two leaves were excised from each seedling and placed on a tray on two pieces of wet paper towels. For inoculation, seven to eight agar plugs from the margin of fresh *R. solani* growth on 1/4-strength PDA were placed on the adaxial surface of each leaf. Seven to eight non-inoculated agar plugs of 1/4-strength PDA were used as controls. Each tray was covered with a lid and incubated on lab bench at ambient temperature with light as above.

After 5 days, yellow to necrotic symptoms were noticeable on *R. solani* treated leaves but no symptoms appeared on control leaves. The control and infected patches of the leaf were excised with a sterile scalpel, snap frozen in liquid nitrogen and processed for RNA extraction with the RNeasy Plus Mini Kit in RLC buffer (Qiagen, Inc.). The purified RNA was treated with DNase at 37°C for 30 min, extracted with phenol and phenol: chloroform, precipitated with ethanol, and dissolved in RNase-free water.

### Construction of Genomic and RNA Libraries and Sequencing

For making genomic libraries, 500 ng of DNA from each sample was sheared with a Covaris sonicator (Covaris E series, Covaris, Inc., Woburn, MA, United States) and paired-end libraries were prepared for sequencing using an Illumina’s HiSeq 2000 platform (Illumina, Inc., San Diego, CA, United States). From end repair until adapter ligation and purification steps of the paired-end, libraries were prepared using the protocol “Illumina library prep” on the IP-Star automated platform (Diagenode IP Star, Diagenode, Inc., Denville, NJ, United States) as per the manufacturer’s protocol. Post ligation, manual protocols were used for gel size selection and PCR amplification using the standard Illumina PCR Cycle (Kapa high-fidelity master mix). The prepared libraries were analyzed on a bioanalyzer and quantified using Qubit (Thermo Fisher Scientific). The normalized libraries were pooled for sequencing (insert size of 500 bp) and submitted for HiSeq 2000 sequencing at the Bioscience Core Laboratory of King Abdullah University of Science and Technology.

Strand-specific mRNA sequencing was performed from total RNA using TruSeq Stranded mRNA Sample Prep Kit LT (Illumina, Inc.) according to the manufacturer’s instructions. Briefly, polyA+ mRNA was purified from total RNA using oligo-dT dynabead selection. First strand cDNA was synthesized using randomly primed oligos followed by second strand synthesis where dUTPs were incorporated to achieve strand-specificity. The cDNA was adapter-ligated and the libraries amplified by PCR. Libraries were sequenced in an Illumina Hiseq 2000 with paired-end 100 bp read chemistry.

### *De novo* Assembly, Genome Annotation and Bioinformatic Analysis

#### Data Preprocessing

Adapter sequences in genomic reads in FASTQ format were trimmed using the trimmomatic tool (version 0.35) (Bolger et al., 2014), followed by trimming low-quality bases at read ends. Read quality was evaluated using the fastqc tool (version 0.11.8) (Simon Andrews, 2020). Reads with length < 20 bp and average quality score < 30 were removed. For genome heterogeneity analysis, *k-mer* distribution analysis on resulting DNAseq reads was performed using jellyfish (version 2.2.10) (Marçais and Kingsford, 2011), which estimated best *k-mer* length for each genome. Histogram distributions of different *k-mers* for the best *k-mer* length were plotted using the *-histo* module of the jellyfish program. In addition, the available raw RNAseq paired-end reads (Supplementary Table 2) were quality trimmed and preprocessed using the same approach used for DNAseq reads. The quality trimmed reads were then subjected to *de novo* assembly using Trinity which predicted transcript sequences (Grabherr et al., 2011). These assembled RNAseq transcripts were used for genome assembly, scaffolding and gene prediction purposes.

#### Genome Assembly

Quality trimmed reads were subjected to *de novo* genome assembly using SPAdes (version 3.7.0) in which a defined range of *k-mer* lengths (21, 33, 55, 65, 77, 101, and 111) was used for contig formation (Bankevich et al., 2012). Quast (version 4.5) was used for quality evaluation of predicted contigs (Gurevich et al., 2013). Scaffolds were subsequently predicted from contigs using SSPACE (version3.0) (Boetzer et al., 2011) and gaps in assembled scaffolds filled using five consecutive runs of GapCloser (version 1.12) (Luo et al., 2012). For samples with a RNAseq dataset available, genome scaffolding was further improved using the Rascaf program (Song et al., 2016). Genome quality was evaluated with BUSCO (version 3.0.1) (Seppey et al., 2019) and scaffolds subjected to ITSx (version 1.1) (Bengtsson-Palme et al., 2013) for ITS sequence prediction. Thereafter, a phylogenetic tree was constructed with megax software (Kumar et al., 2018) using the neighborhood joining method (10,000 bootstraps), in which ITS2 sequences were aligned using ClustalW (Rédei, 2008). The resulting tree was saved in the newick format and visualized together using Phylogeny.IO (Jovanovic and Mikheyev, 2019) and ETE toolkit (Huerta-Cepas et al., 2016). Redundans python script was then used to predict the homozygous genome by reducing the unwanted redundancy to improve draft genome quality (Pryszcz and Gabaldón, 2016). Resulting scaffolds were aligned with mitochondrial genomes of *R. solani* and other Basidiomycota using the blastn program (version 2.6.0; *e*-value ≤1e^–5^) (Camacho et al., 2009) and mapped mitochondrial contigs were removed to retain only the nuclear genome for subsequent annotation.

#### Genome Annotation

The draft genome was annotated using the MAKER (version 2.31.8) pipeline (Cantarel et al., 2008), which predicted intron/exon boundaries, transcript and protein sequences. For the annotation, repeat regions were masked using RepeatMasker (version 4.0.5; model_org = fungi) (Tarailo-Graovac and Chen, 2009). Protein homology evidence was taken from UniProt protein sequences (Reviewed; family: Basidiomycota) (Bateman et al., 2017). For EST evidence, RNAseq reads were assembled into transcripts using Trinity *de novo* assembler (version 2.0.6) (Grabherr et al., 2011). For genomic datasets without corresponding RNAseq datasets available, the EST sequences of alternate organisms were used from previously published *R. solani* genome annotations viz. AG1-IA (Nadarajah et al., 2017), AG1-IB (Wibberg et al., 2015a), AG2-2IIIB (Wibberg et al., 2016b), AG3-Rhs1AP (Cubeta et al., 2014), AG3-PT isolate Ben-3 (Wibberg et al., 2017) and AG8 (Hane et al., 2014). The functional domains, PANTHER pathways (Mi and Thomas, 2009) and Gene Ontology (GO) terms (Ashburner et al., 2000) in the predicted protein sequences were assigned using the InterProScan (version 5.45–80.0) standalone program (Quevillon et al., 2005). The functional domains assigned to each protein included the information from ProSiteProfiles (Hulo et al., 2004), CDD (Marchler-Bauer et al., 2013), Pfam (Finn et al., 2014) and TIGRFAMs (Haft et al., 2003), resulting in the annotated genome in GFF3 format using iprscan2gff3 and ipr_update_gff programs (Quevillon et al., 2005).

We also identified the predicted secreted proteins in each of the *R. solani* proteomes using signalp (version 5.0) (Almagro Armenteros et al., 2019). For identification of proteins with a transmembrane domain, phobius (version 1.01) (Käll et al., 2007) was used. We used targetp (version 1.1) to predict proteins with mitochondrial signal peptides (Emanuelsson et al., 2007). However, since we already removed mitochondrial contigs from assembled genomes, we did not observe any proteins with a mitochondrial signal peptide. Effector proteins in each *R. solani* secretome were predicted using effectorP webserver (version 2.0) (Sperschneider et al., 2016). The Carbohydrate Active enZymes (CAZyme) in *R. solani* proteomes were predicted using dbCAN2 webserver, in which only the proteins predicted by at least two prediction methods were considered (Zhang et al., 2018). The CAZyme family predicted by HMMER was used for the selected proteins.

#### Orthology

Orthologous proteins across all proteomes were identified with orthoMCL clustering using the Synima program (Li et al., 2003; Farrer, 2017), which identified core, unique and auxiliary regions in each *R. solani* proteome. This program was also used for predicting genome synteny using inter-proteome sequence similarity. ShinyCircos was used for circular visualization of synteny plots (Krzywinski et al., 2009; Yu et al., 2018).

### RsolaniDB Database Development

The RsolaniDB (RDB) database was built to host *R. solani* reference genomes, transcript and protein sequences in FASTA format, along with genome annotations included in GFF3 format. For each genome, the information in the database was structured as entries, in which each entry included a list of details about a given transcript and protein, i.e., intron-exon boundaries; predicted functions; associated pathways and GO terms; predicted sequences; orthologs and functional protein sequence domains predicted from InterPro, PrositeProfile and Pfam. The identifier format for each entry (i.e., RDB ID) starts with “RS_” and AG subgroup name followed by a unique number. We also included five previously published *R. solani* annotated genome sequences (i.e., AG1-IA, AG1-IB, AG2-2IIIB, AG3-PT and AG8) with their gene identifiers converted into the RDB ID format. The database was written using DHTML and CGI-BIN Perl and MySQL language, to allow users to perform lists of tasks, including a text-based search for the entire database; or in an AG-specific manner. We also included a list of tools to assist users in performing a number of down-stream analyses, including RDB ID to protein/transcript sequence conversion; FASTA sequence-based BLAST search on the entire database or in an AG-specific manner; a tool to retrieve orthologs for a given set of RDB IDs along with tools for functional enrichment analysis. The GO-based functional enrichment tool for gene set analysis of given RDB IDs was built using the topGO R package (Alexa and Rahnenführer, 2009). Whereas the pathway-based gene set analysis was developed to predict significantly enriched PANTHER pathway IDs for a given set of RDB IDs.

## Results

### Genome-Wide Comparative Analysis of *Rhizoctonia solani* Assemblies and Its Annotation

**FIGURE 1:**
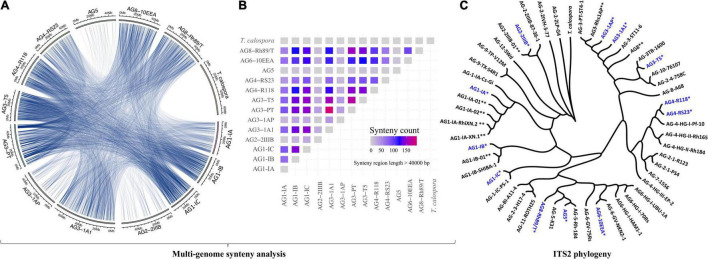
**(A)** Circos plot. The Circos plot represents the syntenic relationship between genomes of different AGs of *Rhizoctonia solani* Kühn. Each line represents the region of genomic similarity predicted with Synima. Only the regions with coverage > 40,000 bases were enumerated and shown. **(B)** The plot highlights the number of high-similarity syntenic regions (coverage > 40,000 bp) shared between each pair of genomes, including *T. calospora*. The red connection represents corresponding isolates sharing a comparatively large number of syntenic relationships relative to other pairs of isolates. Here, self-hits were removed or not shown. **(C)** ITS2 phylogeny. ITS2 sequences of the tester strain were obtained from the NCBI database and were clustered with ITS2 sequences from assembled *R. solani* genomes (highlighted with blue color and *), along with ITS2 sequences from previously published *R. solani* genome assemblies (marked with **). The phylogenetic tree was constructed using megax software with 10,000 bootstrapping steps (see section “Materials and Methods”), after which the resulting tree and corresponding alignment were visualized together using Phylogeny.IO.

We performed high-depth sequencing, *de novo* genome assembly and annotation of 12 *R. solani* isolates. For qualitative evaluation of these assemblies, we used genome sequences of a basidiomycetous mycorrhizal fungus *Tulasnella calospora* (Joint Genome Institute fungal genome portal MycoCosm)^1^ and *R. solani* AG3-PT as negative and positive controls, respectively. Overall, the draft genome assemblies of the *R. solani* isolates showed remarkable differences in genome size, ranging anywhere from the smaller AG1-IC (∼33 Mbp) to the larger AG3-1A1 (∼71 Mbp) isolate genomes (Supplementary Table 3). The number of contigs generated were also highly variable ranging from 678 to 11,793, in which the newly reported assemblies of AG1-IC and AG3-T5 had the highest N50 lengths of 100,597 bp and 196,133 bp, respectively (Supplementary Table 3). The heterogeneity in genomic reads was predicted by analyzing the distribution of different *k-mers* in *R. solani* genomic sequencing reads. The analysis revealed a shoulder peak along with the major peak in *k*-mer frequencies for AG2-2IIIB, AG3-1A1, AG3-1AP and AG8, indicating the possible heterogeneity of these genomic reads of these isolates (Supplementary Figure 1). The G+C content ranged from 47.47 to 49.07%, with a mean of 48.43% (Supplementary Table 3). The quality of these draft genomes was evaluated using BUSCO with scores ranging between ∼88 and 96% (Supplementary Table 3), indicating the completeness of essential fungal genes in the predicted assemblies. Among the presented draft genome sequences, a large number of syntenic relationships (Figure 1A) were identified (length > 40,000 bp), wherein all the given isolates shared at least four highly similar syntenic regions, except *T. calospora* (outgroup), which did not share any syntenic regions with *R. solani* isolates for the given threshold of > 40,000 bp (Figure 1B). The isolates from AG5, AG2-2IIIB and AG3-1A1 shared comparatively lower syntenic regions, whereas AG3-PT (positive control) shared the highest number of syntenic regions with other *R. solani* isolates. In fact, most of the closely related AGs shared a large number of syntenic relationships, e.g., high similarity among AG3 sub-groups. Overall, the analysis exhibited the first line of evidence that indicates widespread collinearity and regions of large similarity across genetically distinct isolates, with *T. calospora* as an outlier.

Subsequently, we performed the ITS2-based phylogeny to compare the ITS2 sequences of the 12 newly sequenced *R. solani* isolates with that of the known *R. solani* tester strains (as positive controls) and *T. calospora* as an outgroup (Figure 1C). We were not able to predict ITS sequences for AG3-PT. Phylogenetic clusters of AGs reflected strong similarity in ITS2 sequences of assembled genomes with that of tester strains of *R. solani*. For instance, the AG1-IA cluster includes four strains, all belonging to the same AG, i.e., AG1-IA. Similarly, ITS2 sequences of different AG3 and AG4 subgroups were clustered within their respective clades, whereas the outgroup *T. calospora* showed distinct architecture, providing strong evidence for correct methods being used here for genome assemblies. Intriguingly, ITS2 sequences of the AG8 subgroup showed remarkable differences, where sequence of the tester strain (i.e., AG-8-A68), previously published genome sequence (i.e., AG8-01) and the genome reported here (i.e., AG8-Rh89/T) were clustered across different clades of the phylogenetic tree.

### Comparison With Other *Rhizoctonia solani* Assemblies

To evaluate the reliability of the genome assemblies, we compared our draft genomes with previously published assemblies of *R. solani* isolates, i.e., AG1-IA, AG1-IB, AG2-2IIIB, and AG8 (Supplementary Figure 2; Cubeta et al., 2014; Hane et al., 2014; Wibberg et al., 2015a,2016b,^2017^; Nadarajah et al., 2017; Ghosh et al., 2019; Lee et al., 2021). The mummer plot (Marçais et al., 2018) comparison showed the overall co-linearity and high similarity among similar assemblies, wherein AG8 assemblies were least co-linear, possibly due to the heterokaryotic nature of the AG8 genome (Cubeta et al., 2014; Hane et al., 2014). In addition to the above representative assemblies, we found several whole genome assemblies of different AG1 subgroups isolates (Nadarajah et al., 2017; Ghosh et al., 2019; Lee et al., 2021). Among these isolates, we selected two representative assemblies, i.e., AG1-IA-XN.2 (96 scaffolds; NCBI accession: GCA_015342405.1) and AG1-IA-RhiXN.1 (16 chromosomes; NCBI accession: GCA_016906535.1), for comparison with our AG1-IA assembly (Supplementary Figure 3). Wherein, the selection of AG1-IA assemblies for comparative analysis is based on the large N50 and genome length of these previously published assemblies. The comparative analysis reveals that, despite the sparse scaffolding of AG1-IA, all the AG1 subgroups shares a large number of syntenic relationships (Supplementary Figures 3A,B). To gain insight into functional domains of these assemblies, we predicted and compare the InterPro domains of all the AG1 isolates under comparison (Supplementary Figure 3C). Interestingly, all the isolates share a significantly large proportion of functional domains, in which AG1-IA shares more than 70% of the domains with at least one of the three other assemblies. Wherein, all the AG1 assemblies possess WD40 repeats domain with a highest proportion (∼9–10%) as compared to the other Interpro domains (Supplementary File 1). Along with a large number of conserved domains, a small percentage of unique functional domains among these assemblies reflects the potential host-specific or isolate-specific functional regions of these assemblies. As expected, the proteomes of these AG1 isolates also shares a large number of orthologous protein clusters (Supplementary Figure 3D), including AG1-IA proteome, which indicates the comparable functional profiles of these assemblies, motivating us to include AG1-IA for further comparative analysis with other sub-groups sequenced in this study.

### Genome-Wide Orthologous Protein Clustering and Functional Analysis

**FIGURE 2:**
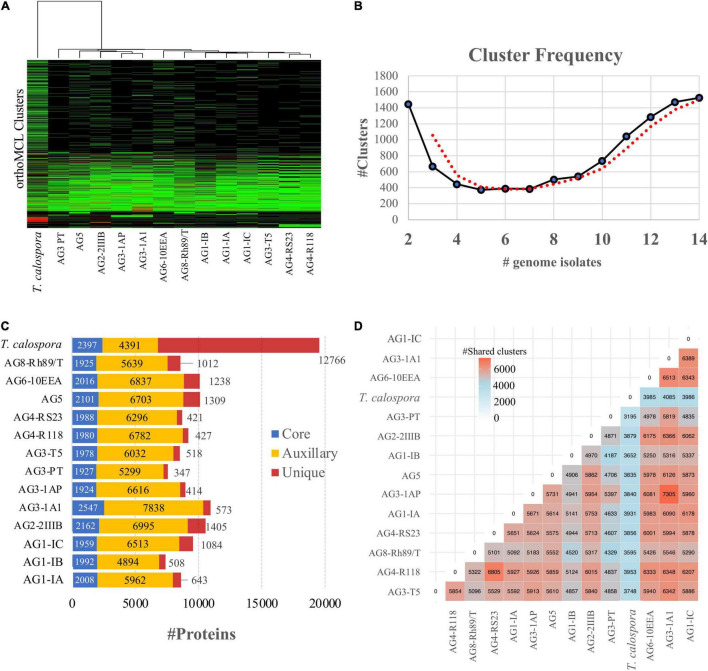
OrthoMCL clustering of the predicted proteomes in *R. solani* AGs. **(A)** Heatmap showing protein conservation across all sequenced *R. solani* AGs and *T. calospora*. Each row represents one orthoMCL cluster, and color is proportional to the number of protein members shared within a given cluster from the given species (black: no member protein present; red: large number of protein members present). The hierarchical clustering (hclust; method: complete) analysis enumerates the similarities between different fungal isolates based on proteins shared by them across all orthoMCL clusters. **(B)** Cluster frequency. The line plot represents the number of orthoMCL clusters shared by different fungal isolates used in this study. Example, > 1,400 orthoMCL clusters are shared by 14 different fungal isolates (including positive and negative controls) used in this study. The bimodal nature of the plot represents high similarities across independent proteomes as large numbers of clusters share protein members from 13 fungal isolates. The red line represents the smoothed curves after averaging out the number of clusters. **(C)** Protein classification based on the orthoMCL clusters. The “core” proteins represent the sub-set of proteomes (from each *R. solani* AG and *T. calospsora*) with a conserved profile across all the isolates. Similarly, the “unique” sets represent the isolate-specific protein subset. The rest of the protein subsets make up the “Auxiliary” proteome which are conserved in a limited number of isolates. **(D)** Shared orthoMCL clusters. The number of orthoMCL clusters shared between any two isolates. A shared cluster means, a given orthoMCL cluster contains proteins from both the isolates.

Intron/exon and transcript boundaries were identified using the maker pipeline (see section “Materials and Methods”), which predicted 7,394–10,958 protein coding transcripts per genome (excluding *T. calospora*, Supplementary Figure 4) in which the AG3-1A1 genome had the highest number of transcripts. Next, using OrthoMCL, the translated protein sequences in all genomes were clustered into orthologous groups, where each cluster of proteins represented a set of similar sequences likely to represent a protein family. The similarities among the given isolates were enumerated by measuring proteins shared by different proteomes in the same orthoMCL clusters (Figure 2A). As expected, this analysis clearly outgrouped *T. calospora*, indicating that it has a different protein family composition than *R. solani* isolates. The AG1 and AG4 subgroups, AG3-1A1 and AG3-1AP, showed expected similarities and shared similar clustering profiles while AG3-PT and AG5 showed a divergent profile of protein families with respect to the other AGs studied. A large set of orthoMCL clusters shared proteins from all/most of the *R. solani* isolates which further indicates inherent similarities as well as unique attributes across these pathologically diverse groups of fungi. For instance, more than 1,400 orthoMCL clusters were composed of proteins belonging to only two AGs, whereas > 1,500 clusters were composed of proteins from all 13 *R. solani* isolates (including previously reported AG3-PT) and *T. calospora* (Figure 2B). It is expected that these conserved clusters are composed of proteins from core gene families with essential functions, whereas other clusters may contain proteins with unique AG-specific roles (Figure 2C). The analysis revealed that AG1-IC, AG2-2IIIB, AG5, AG6-10EEA and AG8 were composed of a large number of unique proteins (>1,000 proteins), whereas AG3-1A1 had the highest number of core and auxiliary proteins. The pair-wise comparison of the number of clusters shared by any two AGs highlighted that AG3-1AP shares the highest number of orthoMCL clusters with AG3-1A1, a sector derived hypo-virulent isolate of AG3-1AP (Figure 2D; Lakshman et al., 1998). In fact, AG3-1A1 proteins shared a large number of clusters with other AG subgroups too, including AG1-IC, AG6-10EEA, AG2-2IIIB and AG4-R118.

**FIGURE 3:**
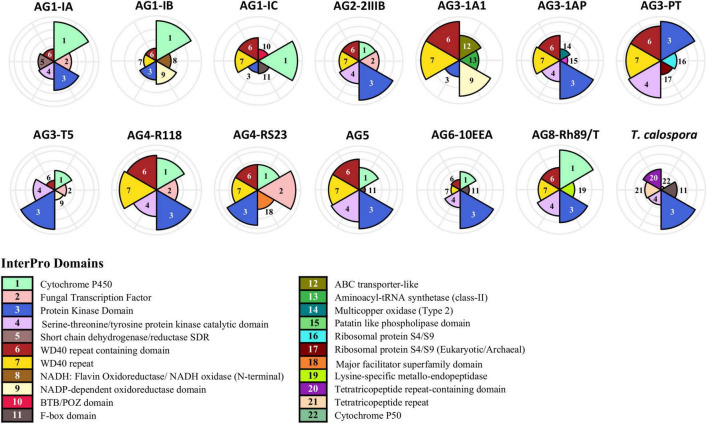
InterPro domain analysis of the unique proteome. In the unique proteome of each fungal isolate, InterPro protein domain families were predicted using InterProScan (Version 5.45–80.0). Only the top five most enriched protein families are shown. The number marks the corresponding annotation of InterPro family domain in the circular bar plot.

To investigate the functional composition of proteins using orthologous groups, we performed InterPro domain family analysis of proteomes from each AG (Supplementary Figure 5). The core proteome of most AGs was composed of ∼2,000 InterPro domain families, whereas the unique proteome per AG ranged between 101 (for AG3-PT) to 628 (for AG3-1A1) domain families. The most common protein family in the unique proteome of *R. solani* subgroups was “Cytochrome P450,” which is essential for fungal adaptations to diverse ecological niches (Črešnar and Petrič, 2011; Figure 3). Proteins with WD40 repeats were found to be the most common in the unique proteome in most AGs. A few of the AG subgroups were found to be enriched with a protein family that was significantly associated with its unique proteome only, possibly being involved in the survival of that AG in respective hosts. For instance, the AG1-IB unique proteome was enriched with “NADH: Flavin Oxidoreductase/NADH oxidase (N-terminal),” similarly AG3-1A1 was enriched with “ABC transporter-like” and “Aminoacyl-tRNA synthetase (class-II)” InterPro domains. Likewise, AG3-PT was found to be uniquely enriched with “Ribosomal protein S4/S9” and AG3-1A1 was uniquely enriched with “Multicopper oxidase (Type 2)” and “Patatin like phospholipase domain.”

### A Large Proportion of Protein Clusters Are Uniquely Enriched in *Rhizoctonia solani* Proteomes

The unique components of the 12 proteomes associated with this study and their attributes were compared to the six previously reported *R. solani* spp. (i.e., AG1-IA, AG1-IB, AG3-PT, AG3-Rhs1AP, AG3-2IIIB and AG8). The 18 proteomes with a total number of 180,491 protein sequences (including 6 previously reported *R. solani* spp.) were clustered using CD-HIT (similarity threshold = 65%). CD-HIT enumerated 60,441 protein clusters, of which 46,804 clusters (77.43%) included at least one of the protein sequences from the previously reported *R. solani* proteome. Whereas 22.56% of the clusters (i.e., 13,637 non-identical CD-HIT clusters) were only associated with one or more of the 12 *R. solani* isolates reported in this study. The analysis suggests 15.6% (*n* = 18,542 protein sequences) of the total sequences (i.e., *n* = 118,812 from 12 *R. solani* isolates) generated in this study were sequentially non-identical to previously published proteomes, possibly providing novel sequences and functional information for hypothesis development. Therefore, we investigated the Interpro domains predicted in the protein sequences of 13,637 non-identical CD-HIT clusters which elucidated 3,931 unique Interpro domain IDs, among which most enriched protein domains belonged to the WD40 repeat family, protein kinase domain, F-box domain and Zn(2)-C6 fungal type DNA binding domain. Overall, among the 3,931 Interpro domains, 128 domains were not reported in any of the six previously published genomes/proteomes and were exclusively associated with the 12 *R. solani* isolates reported here. The complete list of non-identical CD-HIT clusters along with their protein components, enriched Interpro domains (128 unique and complete list of 3,931 Interpro domain IDs) are available in Supplementary File 2.

### Predicted Secretome and Effector Proteins

**FIGURE 4:**
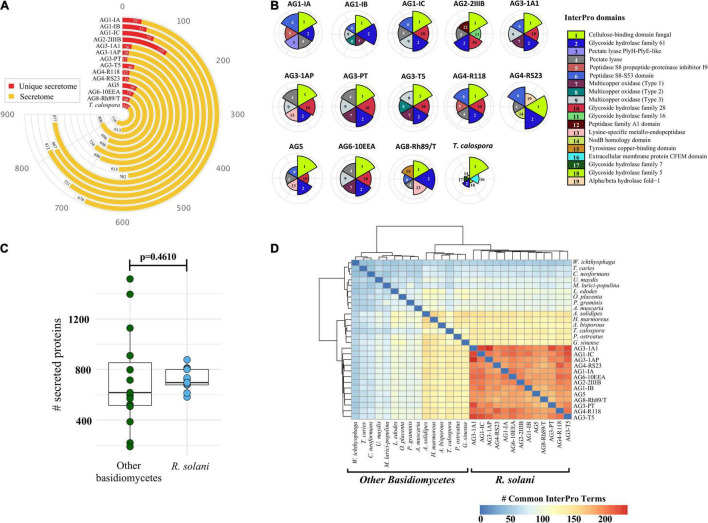
The secreted proteins. **(A)** Number of predicted proteins in the secretome of each fungal isolate (highlighted in yellow). The secreted proteins predicted in the unique proteome of each isolate is highlighted in red. **(B)** Comparative analysis of the top six highly enriched InterPro domains in the secretome. **(C)** Comparative analysis of the total number of secreted proteins predicted in *R. solani* isolates as compared to other basidiomycetes used in this study. *P*-value is computed using the unpaired Wilcoxon-rank sum test. **(D)** Heatmap showing the pairwise comparison of the InterPro terms commonly shared by the secretome of *R. solani* isolates as well as other basidiomycetes. The strong hierarchical clustering of R. solani isolates highlights their functionally unique and distinct secretome profile as compared to other basidiomycetes.

To facilitate host colonization, plant pathogens secrete proteins to host compartments to establish fungal infection (Kim et al., 2016; McCotter et al., 2016; Li et al., 2019). Therefore, we identified the comprehensive set of secreted proteins from the 12 *R. solani* genomes and the *T. calospora* genome. Figure 4A shows the number of secreted proteins identified in each of the genomes, where AG1-IC, AG3-1A1, AG6-10EEA and, AG2-2IIIB contained a large number of proteins in the predicted secretome (Supplementary File 3,Sheets 1,2). Isolates from AG1-IC, AG2-2IIIB and AG8 contained a comparatively larger number of isolate-specific secreted proteins (i.e., secreted proteins in the unique proteome), while isolates from AG3-1AP, AG3-1A1 and AG3-PT contained a comparatively lower number of secreted proteins. Interestingly, InterPro domain analysis of the secreted proteins suggested that the most enriched protein domain in the predicted secretome was “cellulose binding domain—fungal” (Figure 4B) which is essential for degradation of cellulose and xylans (Linder et al., 1995). In addition, the secretomes were also enriched with proteins containing “Glycoside Hydrolase Family 61,” “Pectate Lyase” and “multi-copper oxidase family” domains. Most of these protein components function in degradation of the plant host cell wall and breaking down the first line of host defense. We observed that certain families of protein domains were enriched within a few AGs only. For instance, “aspartic peptidase family A1” domain containing proteins, involved in diverse fungal metabolic processes, were mainly enriched in the AG2-2IIIB isolate, similarly the “lysine-specific metallo-endopeptidase” domain was enriched in AG3-1AP, AG5 and AG8. The AG4-R118 secretome was significantly enriched with proteins belonging to “Glycoside Hydrolase Family 28” and “Peptidase S8 propeptide-proteinase inhibitor I9” domains, whereas the AG4-RS23 secretome was composed of “NodB homology” and “alpha/beta hydrolase fold-1” domains. Taken together, the analysis indicated that each of the given AG secretomes was significantly enriched with a unique set of protein families that possibly allows a variety of biological functions in different host systems.

To further identify unique and conserved attributes associated with all 13 *R. solani* isolates (including previously published genomic information from the AG3-PT isolate, as positive control), we performed a comparative analysis of their secretomes with the secretomes of 14 other fungi (excluding *T. calospora*), which represented the major taxonomic, pathogenic, ecological, and commercially important (edible fungi) groups within the Division Basidiomycota (Supplementary Table 4). We hypothesized that a small set of functionally important proteins, e.g., secreted proteins, in *R. solani* may have unique attributes not observed within the other Basidiomycetes. The number of secreted proteins predicted in *R. solani* AGs were not significantly different with the number of secreted proteins in other Basidiomycetes (*p* = 0.0629; Figure 4C). However, the InterPro domains enriched in the secretome of *R. solani* AGs and other Basidiomycetes were found to be significantly different. Only a limited number of InterPro terms were shared between *R. solani* AGs and other Basidiomycetes, and *R. solani* AGs were functionally closer to each other than to the other Basidiomycetes (Figure 4D), suggesting that *R. solani* secretomes have a unique domain profile. Overall, 565 InterPro terms were found in the secretome of *R. solani*, whereas the other Basidiomycete (including *T. calospora*), secretomes were enriched with 620 terms where 283 InterPro terms were common across both groups of species. There were 282 InterPro terms (50%) uniquely associated with *R. solani*, not observed in the secretome of other Basidiomycetes, and 337 InterPro terms only observed in the secretome of other Basidiomycetes. The *R. solani*-specific 282 InterPro terms included several protein domains belonging to diverse functional groups, e.g., “Aspartic peptidase A1 family,” “Cysteine rich secretory protein related” and “Polyscaccaride lyase 8” domains. Among the domains commonly enriched across both *R. solani* isolates and other Basidiomycetes, we calculated the fold change of difference of domain occurrence in their secretome and enumerated the protein domains with significant differences in *R. solani* as compared to the other Basidiomycetes (Supplementary File 3, Sheets 1,2). The analysis suggested that proteins with domains like “Pectate lyase,” “Serine amino-peptidase” and “Lysine-specific metallo-endopeptidase” were significantly enriched in *R. solani* secretomes. Similarly, proteins with “Hydrophobin” and “Zinc finger ring-type” domains were enriched in the other Basidiomycetes. We believe that the large number of unique functional domains in the secreted proteome of *R. solani* may be functionally relevant, allowing these fungi to survive under diverse conditions, and should be investigated to understand their role in survival.

**FIGURE 5:**
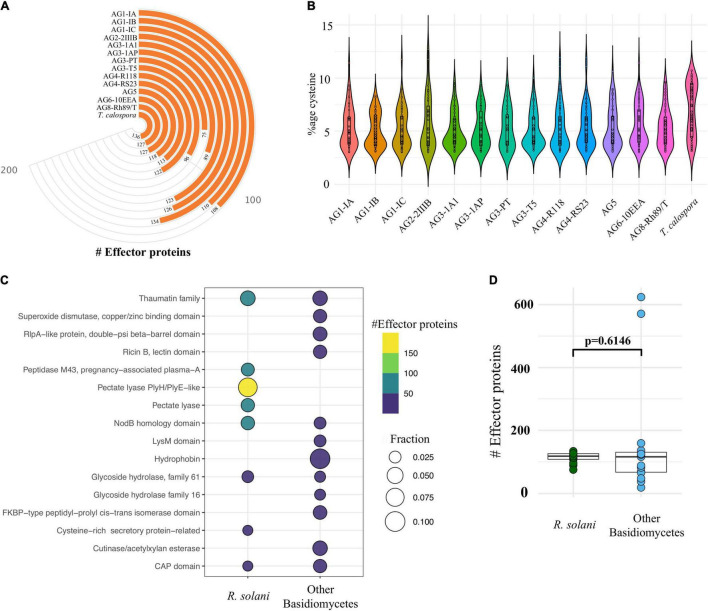
Effector proteins. **(A)** The number of cysteine rich effector proteins predicted in the predicted secretome of each fungal isolate. **(B)** The proportion of cysteine observed across all the effectors predicted in each isolate. **(C)** Topmost enriched InterPro domains in effector proteins of *Rhizoctonia* species (not *T. calospora*) and other Basidiomycetes (including *T. calospora*). **(D)** The comparative analysis of the distribution of number of effector proteins predicted in *R. solani* AGs as compared to other Basidiomycetes. The *p*-value is computed using the unpaired Wilcoxon-rank sum test.

Although these plant pathogenic fungi secrete a large number of proteins, only a small proportion have been implicated in fungal-plant disease interactions, i.e., effector proteins (Kim et al., 2016; McCotter et al., 2016; Li et al., 2019). Effector proteins can strongly inhibit the activity of host cellular proteases and allow pathogenic fungi to evade host defense mechanisms. Fungal effector proteins are not known for having a conserved family of domains, these proteins typically being of small length (300–400 amino acids) and higher cysteine content (Stergiopoulos and de Wit, 2009; McCotter et al., 2016; Sperschneider et al., 2016). Our analysis revealed 75–134 predicted effector proteins in *R. solani* genomes, whereas *T. calospora* contained 136 effector proteins (Figure 5A).

Isolates from AG1-IC contained the highest number of effector proteins (*n* = 134), whereas the isolate from AG3-PT contained a small number of effectors (*n* = 75). On average, isolates contained approximately 100 effector proteins which had a similar proportion of cysteine residues (Figure 5B). The topmost enriched domain among all *R. solani* effector proteins was “Pectate lyase” followed by “thaumatin family” of domain containing proteins (Figure 5C; Supplementary File 3,Sheet 3).

The other Basidiomycetes studied were enriched with a similar number of effector proteins (*p* = 0.14; Figures 5C,D). Effector proteins in *R solani* AGs included proteins belonging to 237 InterPro terms, whereas effector proteins from the other Basidiomycetes (including *T. calospora*) included proteins enriched with 119 terms. We found 173 terms (72%) that were uniquely associated with *R. solani* AGs, in which most abundant terms include IPR001283 (Cystine-rich-secretory-protein related) (Supplementary File 3, Sheet 4). These unique effectors may play the deciding roles in host recognition and virulence of *Rhizoctonia* pathogens (Yamamoto et al., 2019; Wei et al., 2020). Also, 64 InterPro terms were commonly enriched in both groups of effector proteins, wherein “Pectate lyase” and “Glycoside hydrolase family 28” were mainly associated with *R. solani* AG subgroups and “Hydrophobin” was mainly associated with other Basidiomycetes. The complete list of secretome, effector proteins, InterPro domains and associated information are available in Supplementary File 3. The complete list includes those predicted secretome and effector proteins, that are already known to be associated with the *Rhizoctonia* secretome (e.g., Ricin domain) but not highlighted above.

### Carbohydrate-Active Enzymes

**FIGURE 6:**
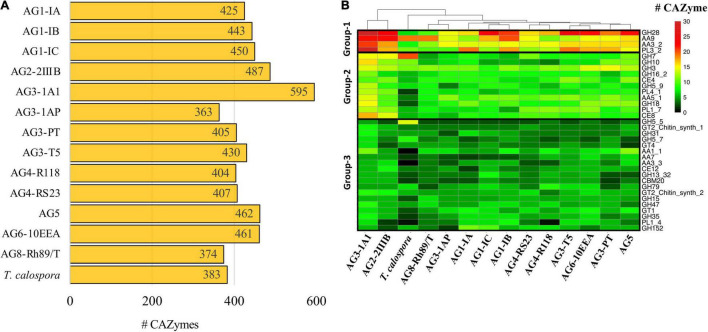
CAZymes**. (A)** The number of carbohydrate-metabolizing enzymes (CAZymes) predicted in the proteome of each fungal isolate. **(B)** Heatmap showing the CAZyme conservation across all the *R. solani* AGs and *T. calospora*. Each row represents one CAZyme family of proteins, and color is proportional to the number of protein members shared within a given family from the given species (black: no member protein present; red: large number of protein members present). The hierarchical clustering (hclust; method: complete) enumerates the similarities between different fungal isolates based on proteins shared by them across all CAZyme families. For simplicity only the CAZyme families enriched in more than 50 enzymes across all proteomes are shown.

CAZymes are essential for degradation of host plant cells and fungal colonization in the host (Kameshwar et al., 2019; Barrett et al., 2020). Using CAZy (Carbohydrate Active Enzyme database) (Lombard et al., 2014), which contains classified information regarding enzymes involved in complex carbohydrate metabolism, we annotated and compared the distribution of CAZymes in all *R. solani* isolates. Overall, *R. solani* isolates were composed of 383–595 high confidence CAZymes, with AG3-1A1 having the largest number of CAZymes (Figure 6A). These predicted CAZymes in *R. solani* AGs were mainly distributed across 177 CAZyme families that can be broadly classified into six major classes of enzymes, i.e., Glycoside Hydrolase (GH), Polysaccharide Lyase (PL), Carbohydrate Esterase (CE), Carbohydrate-binding modules (CBM) and redox enzymes with Auxiliary Activities (AA). Our analysis revealed that GH forms the major class of CAZymes in all fungal species, including *T. calospora* (Supplementary Figures 6, 7); this enzyme hydrolyzing glycosidic linkages between carbohydrate and non-carbohydrate moieties or two or more carbohydrate moieties (Henrissat, 1991). Whereas CBM forms the least abundant class of enzymes enriched in the proteomes of the given isolates. Despite the differences, we observed a similar distribution of the enzyme count in each class of CAZyme across all given isolates.

Among the predicted 177 families, only 34 families were abundant (with total enzyme count > 50 proteins; Figure 6B) across all the given isolates, i.e., *Rhizoctonia* species and *T. calospora*. These 34 families had a distinct abundance profile in each AG, for instance, proteins from the GH7 family was highly abundant in *T. calospora* as compared to the *R. solani* isolates. Similarly, proteins belonging to PL1_4 were not observed in AG4-R118 and *T. calospora*. We divided these 34 families into three different groups, with respect to their abundance profile in *R. solani* isolates. Group-1 contained CAZymes belonging to GH28, AA9, PL3_2 and AA3_2 families and formed the highly abundant families (total enzyme count > 200 proteins) of enzymes in *R. solani* AGs. Similarly, Group-2 contained 11 CAZyme families with enzymes moderately abundant in *R. solani* AGs. Whereas Group-3 contained 19 families with sparsely abundant CAZymes. In all three clusters, AG3-1A1 contained the highest number of CAZymes for most of the 34 families and was significantly enriched with all members of Group-1 families. In fact, the clustering analysis highlighted the similar profiles of AG3-1A1 and AG2-2IIIB, mainly due to a similar distribution of proteins belonging to GH28, AA9, AA3_2 and GH7. In Group-1, although GH28 containing enzymes were abundant in most of the *R. solani* isolates, AG8 contained a limited number of enzymes belonging to this family. Similarly, AA9 and PL3_2 families of enzymes were abundant only in 50% of the isolates and may provide a unique set of functions to the respective isolates. In Group-2 there was a similar distribution of abundance profiles across all isolates, except *T. calospora*, which indicated their probable role in *R. solani* specific functions. For example, CAZymes belonging to AA5_1, GH18 and PL4_1 were enriched in most of the *R. solani* isolates, but not in *T. calospora*. The conserved distribution of CAZyme families in the diverse proteomes of different *R. solani* isolates signified their essential role in fungal activity. Group-3 CAZymes provided unique and distinct profiles to each AG with a limited number of families showing a similar abundance profile. Wherein, *T. calospora* was found to be distinctly abundant in CAZymes belonging to GH5_5, not observed with *R. solani* isolates. These results strongly suggested that *R. solani* isolates share a large proportion of carbohydrate degrading enzymes, in which an isolate-specific CAZyme profile can also be observed (mainly from Group-3). To confirm if the abundance profile was strictly associated with *R. solani* isolates, we performed the comparative analysis with the abundance profile from the 14 other Basidiomycetes. The analysis clearly revealed a distinct CAZyme profile from other Basidiomycetes, in which *R. solani* isolates were phylogenetically grouped into a different cluster (Supplementary Figure 8). The analysis highlighted the families that were uniquely abundant in *R. solani* isolates rather than other Basidiomycetes, e.g., GH28, PL3_2, AA5_1, CE4, GH10, GH62, PL4_1, CE8, PL1_7, PL1_4 and AA7, and as expected, most families belonged to Group-1 and Group-2 of the previous analysis. Among these families, PL3_2, GH62 and CE8 were distinctly expressed in *R. solani* isolates.

### RsolaniDB: A *Rhizoctonia solani* Pangenome Database and Its Applications

RDB is a large-scale, integrative repository for hosting the *R. solani* pangenome project with emphasis on supporting data mining and analysis, wherein the genomes and their components can be accessed under three different categories, viz. genomic, ortholog and functional assignment.

#### Genomes

The genomic content includes draft genome sequences of *R. solani* isolates in FASTA format along with the gene level annotation in GFF3 format. The annotation includes prediction of gene boundaries with introns and exons, as well as their locations on contigs or scaffolds. It also includes the predicted transcribed cDNA sequences and translated protein sequences. This information is vital for those users looking for reference genomes and their annotated components for mapping RNAseq reads. The draft genomes and their annotation can also be downloaded and used for downstream local analysis, e.g., variants calling, SNP, eQTLs analysis and other similar genomic analyses with different bioinformatics methods.

#### Orthologs

Using the orthoMCL clustering on the proteomes of 18 *R. solani* (including previously published genome assemblies), protein sequences were compared and clustered into groups of similar sequences. The sequences not part of any of the clusters, i.e., singletons, and unique to respective isolates were categorized as “unique.” Whereas the rest of the proteome was categorized either into “core” or “auxiliary” groups of orthoMCL clusters. RDB allows users to retrieve this information for each protein entry and the protein ID of other members of its ortholog cluster family, if any.

#### Functional Assignment

This category includes the predicted InterPro protein domains associated with each of the protein entries. RDB also includes GO information associated with each protein, along with PANTHER pathway terms. This information helps in assigning the functional description for each protein entry in the database.

The database is organized to include one unique RDB ID (or entry) for each gene structure, with all the above associated information. The RDB ID allows users to search the genomic coordinates (intron/exon boundaries) with IGV visualization, sequences and its functional annotation, for each gene in each *R. solani* isolate. This information can be retrieved from the database via the “text-based” or “keywords-based” search in an AG-specific manner or from the entire database. Users can also perform blast searches of their own nucleotide or protein sequences to the entire database or can target a given AG. Moreover, users can retrieve the set of sequences in FASTA format, for a given list of RDB IDs. One of the important and unique features of RsolaniDB tools allows users to perform functional or gene-set enrichment analysis of given RDB IDs, e.g., Gene Ontology or pathway analysis. This feature is especially useful for analyzing differentially expressed genes after RNAseq data analysis, as it provides the statistical significance (as *p*-values) of different GO/pathway terms enriched in a given set of differentially expressed genes. As far as we know, this feature is unique to RDB with respect to any other existing *Rhizoctonia* resources. However, it requires the user to use reference genome sequences and the annotation file from RDB database for subjecting into the RNAseq data analysis pipeline. As an additional resource, RDB also incorporated previously published (Cubeta et al., 2014; Hane et al., 2014; Wibberg et al., 2016a; Nadarajah et al., 2017) genome and transcriptome level information in a single platform with an RDB ID format. The database is publicly available to the scientific community, accessible at http://rsolanidb.kaust.edu.sa/RhDB/index.html.

## Discussion

*Rhizoctonia solani* is considered one of the most destructive and diverse groups of soil-borne plant pathogens causing various diseases on a wide range of economically important crops. It is classified into 13 AGs with distinctive pathogenic host ranges and responsiveness to disease control measures. Until now, draft genome assemblies belonging to only four of the 13 AGs had been reported; viz. AG1-IA (Nadarajah et al., 2017), AG1-IB (Wibberg et al., 2015a,b), AG2-2IIIB (Wibberg et al., 2016b), AG3-Rhs1AP (Cubeta et al., 2014), AG3-PT isolate Ben-3 (Wibberg et al., 2017), and AG8 (Hane et al., 2014). Here we expanded the scope of genetic analysis of the *R. solani* complex by performing comprehensive genome sequencing, assembly, annotation and comparative analysis of 12 *R. solani* isolates. This enabled us to perform pangenome analysis of *R. solani* on 7 AGs (AG1, AG2, AG3, AG4, AG5, AG6, AG8), selected additional sub-groups (AG1-IC, AG3-TB), and a hypovirulent isolate (AG3-1A1). Although the heterokarotic and diploid nature of *Rhizoctonia* species were expected to cause genome assembly challenges (Wibberg et al., 2016b), we observed a large number of inter-group syntenic regions as well as ITS2-based and protein sequence and domain similarities which highlights the high similarities among the 13 *R. solani* isolates (including AG3-PT) studied. In addition, a large proportion of CD-HIT protein clusters were also found to be conserved in these isolates when compared to the previously reported *R. solani* protein sequences, thus reassuring the data generation, processing pipelines as well as the high quality of the draft genome sequences reported in this study.

To deduce the similarities and unique features of predicted proteomes, we performed a series of comparative analyses that indicated the expected heterogeneity among *R. solani* subgroups with the orchid mycorrhizal fungus *T. calospora* as an outlier. For example, both AG5 and AG2-2IIIB included a large set of unique proteomes as well as secretomes, enriched with InterPro families of proteins that were abundant in these two AGs. Additionally, the proteome of *R. solani* isolates was uniquely and highly enriched with proteins with “pectate lyase” domains, when compared to other Basidiomycetes. Another finding of potential significance was that the highest number of orthoMCL clusters was shared between AG3-1A1 and AG3-1AP, both isolates belonging to the AG3-PT subgroup. Isolate AG3-1A1 is the sector-derived, hypovirulent isolate of the more virulent isolate, AG3-1AP. Intriguingly, AG3-1A1 has been demonstrated to be a successful biocontrol agent of isolate AG3-1AP in the field (Bernard et al., 2012). A high degree of overlap in gene function is consistent with competitive niche exclusion as the mechanism of biocontrol. Overall, by using a novel set of genomes, orthoMCL comparative analysis highlighted several unique relationships across the *R. solani* isolates, that may be used by mycologists in disease management strategies.

Secretome analysis revealed several interesting findings that provided unique characteristics to each *R. solani* isolate, e.g., the secretome of AG1-IB and AG3-T5 being uniquely and significantly enriched with three different multi-copper oxidases (type 1/2/3), isolates from both AGs being known to cause foliar diseases. Despite inherent differences, most of the secretomes had similar composition in their significantly enriched protein domains, which mainly included “Cellulose-binding domain fungal,” “Glycoside hydrolase family 61” and “Pectate lyase.” However, the composition was significantly different with respect to other Basidiomycetes and a large number of reported protein families were uniquely associated with multiple *R. solani* isolates. We observed similar findings for the effector proteins, wherein proteins containing “Cysteine rich secretory proteins,” “Pectate lyase” and “Thaumatin” were distinctly abundant in *R. solani* isolates, while “Hydrophobin” was only abundant in other Basidiomycetes. Similarly, the CAZyme analysis highlighted several unique attributes associated with each *R. solani* species especially AG3-1A1 possessing the CBM1 family of proteins which are linked with degradation of insoluble polysaccharides (Van Bueren et al., 2005). Several families of CAZymes were not present in *T. calospora*, which is a symbiotic mycorrhizal fungus, and other Basidiomycetes, e.g., GH28, PL3_2, AA5_1 and GH10 (Fochi et al., 2017). In addition, AG3-1A1 had exceptionally abundant proteins in AA9 and GH28, a feature not observed with any other Basidiomycetes. In contrast, AA3_2 (Group-1) was abundant in most of the Basidiomycetes, including *R. solani*. Overall, data presented in this study were consistent with the hypothesis that AGs and sub-groups of *Rhizoctonia* species are highly heterogeneous, each with unique functional genomic properties, while being conserved in their functional regions with respect to other groups. The unique secretomes, effector and similarly CAZymes profiles of *R. solani* relative to other Basidiomycetes may reflect the ecological and host adaptation strategies and calls for future research to better understand the biology and pathology of this species complex.

Since each of the *R. solani* AGs or subgroups is characterized by a unique heterogeneous profile, we strongly believe that the presented genome assemblies, annotation and comparative analyses available with our web-resource *viz.* RsolaniDB (RDB) will facilitate mycologists and plant pathologists in generating a greater understanding of *R. solani* biology and ecology, and in developing its disease management projects, including drug target discovery and design of future diagnostic tools for its rapid discrimination under indoor and outdoor farming environments.

## Data Availability Statement

The processed fastq files and the annotated genome assemblies generated and presented in this study can be found in the online repositories. The name of the repositories and accession numbers can be found below: https://www.ebi.ac.uk/arrayexpress/, E-MTAB-9588 https://www.ebi.ac.uk/ena, PRJEB42614.

## Author Contributions

AK, DL, and AP conceived the study, interpreted the results, and wrote the manuscript. AK performed the bioinformatics analysis and developed the computational pipelines and the database. AR, SM, and MN conducted the molecular experiments, library preparation, and sequencing. DR collected and stored the materials and edited the manuscript. AP and DL supervised the overall project. All authors contributed to the article and approved the submitted version.

## Conflict of Interest

The authors declare that the research was conducted in the absence of any commercial or financial relationships that could be construed as a potential conflict of interest.

## Publisher’s Note

All claims expressed in this article are solely those of the authors and do not necessarily represent those of their affiliated organizations, or those of the publisher, the editors and the reviewers. Any product that may be evaluated in this article, or claim that may be made by its manufacturer, is not guaranteed or endorsed by the publisher.

## Footnotes

1
http://genome.jgi.doe.gov/Tulca1/Tulca1.home.htm
