# Supplementary material for: Pangenome Analysis of the Soilborne Fungal Phytopathogen Rhizoctonia solani and Development of a Comprehensive Web Resource: RsolaniDB
Source: Front Microbiol. 2022 Mar 25;13:839524. doi: 10.3389/fmicb.2022.839524 (PMC8992008; doi:10.3389/fmicb.2022.839524)
Supplement: Supplementary file 1 [file Data_Sheet_1.pdf]

Pangenome Analysis of the Soilborne Fungal Phytopathogen *Rhizoctonia solani* and Development of a Comprehensive Web Resource: RsolaniDB

Kaushik, A.[1], Roberts, D.P.[2], Ramaprasad A.[1], Mfarrej, S.[1], Mridul Nair[1], Lakshman, D.K.*[2] and Pain, A.*[1, 3]

[1]Biological & Environmental Science & Engineering Division, KAUST, Thuwal 23955-6900, Saudi Arabia

[2]Sustainable Agricultural Systems Laboratory, USDA-ARS, Beltsville, MD 20705, USA

[3]International Institute for Zoonosis Control; Hokkaido University, Sapporo, 001-0020 Japan

* Corresponding authors

Supplementary information

Supplementary tables

Table S1. Details of *Rhizoctonia solani* cultures used in the genomic investigation

Isolate number	AGs	AG subgroups	Isolate name	Plant Host	Reference	Obtained from
1	AG1	AG1-IA	BM1	Soybean	*in-lab* isolate	BM
2		AG1-IB	BM2	Tall fescue grass	(1)	BM
3		AG1-IC*	BM3	Sugarbeet	(1)	BM
4	AG2	AG2-2IIIB	Rh-146	Bentgrass, Georgia, USA	(1)	LB
5	AG3	AG3-PT	Rhs1AP or 1AP	Potato, Maine, USA (This isolate was sequenced by Cubeta et al and named as Rhs 1AP)	(2,3)	
6			Rhs1A1 or 1A1	Sectored, hypovirulent isolate of Rhs 1AP	(2)	*In lab* isolate
7		AG3-TB*	AG3-T5 or T5	Tobacco, North Carolina, USA	(4)	MC
8	AG4*	HG-I	R118-11	A basidiospore isolate of parent isolate R118 isolated from soil, Maryland, USA	(5)	*American Type Culture Collection, CN* 16118
9			RS23A or Rs23	Soil, Maryland, USA	(6,7)	*In lab* isolate
10	AG5*	N/D	63/T	Tall fescue grass	(1)	LB
11	AG6*	N/D	10EEA	Sweet gum, North Carolina, USA	(1)	MC
13	AG8	N/D	Rh89/T	Oat root, Scotland	(1)	LB

N/D = Sub-groups not identified or does not exist.
BM: Bruce Martin, Clemson University, South Carolina.
LB: Lee Burpee, University of Georgia.
MC: Marc Cubeta, North Carolina State University.
*First genome sequences reported in this study for an AG, sub-group, a sub-group or a secotor-derived hypovirulent isolate.

Table S2: Summary of genome libraries and sequencing reads.

AG subgroups	Isolate number	Library Type	Read Count[#]	GC %	Mean read length	RNAseq available
AG1-IA	BM1	Paired end	~97M	45	86	Yes
AG1-IB	BM2	Paired end	~52M	45	80	-
		Paired end	~65M	46	80	
AG1-IC	BM3	Paired end	~39M	46	76	-
		Mate-pair	~33M	45	76	
AG2-2IIIB	Rh-146	Paired end	~157M	45	85	Yes
		Paired end	~41M	46	82	
		Single end	~41M	46	89	
AG3-1A1	Rhs1A1	Paired end	~105M	44	76	Yes
		Paired end	~37M	47	82	
		Mate-pair	~21M	47	69	
AG3-1AP	Rhs1AP	Paired end	~97M	45	85	-
AG3-T5	*	Paired end	~103M	45	79	Yes
		Paired end	~43M	46	71	
		Mate-pair	~26M	47	81	
AG4-R118	*	Paired end	~44M	46	84	Yes
		Paired end	~37M	46	77	
		Mate-pair	~25M	47	77	
AG4-RS23	*	Paired end	~130M	46	84	-
AG5	63/T	Paired end	~38M	47	76	Yes
		Mate-pair	~27M	48	77	
AG6-10EEA	*	Paired end	~42M	47	76	-
		Mate-pair	~42M	46	75	
AG8	Rh89/T	Paired end	~22M	46	80	-
		Paired end	~48M	46	71	
		Paired end	~37M	45	76	
		Mate-pair	~44M	45	76	

# in millions; QC, trimmed and error corrected reads

* Not available

**Table S3: Assembly properties of genomes** reported in this study								
	Genome Length	GC (%)	# contigs	N50	# N's per 100 kbp	Largest contig length	BUSCO	Repetitive genome (%age)
AG1-1A	46,986,790	47.47	9,145	13,594	10.92	162,406	89.30%	1.47
AG1-1B	34,314,604	48.34	1,833	69,438	3.86	607,833	95.80%	0.68
AG1-1C	33,636,943	48.07	1,221	100,597	800.74	921,812	93.40%	0.56
AG2-2IIIB	57,816,428	48	10,766	11,419	5.53	76,418	92.10%	0.82
AG3-1A1	71,708,704	49.05	7,116	29,147	579.62	485,982	96.50%	0.72
AG3-1AP	46,445,294	48.23	10,166	8,499	7.87	213,864	90.30%	0.78
AG3-T5	35,199,394	48.68	678	196,133	1629.7	79,7852	95.20%	0.75
AG4-R118	38,232,519	48.45	1,860	52,568	597.37	638,355	91.30%	0.65
AG4-RS23	42,733,718	48.22	4,504	19,406	47.48	232,254	89.60%	0.88
AG5	47,761,425	49.01	6,674	12,763	1961.37	85,396	88.70%	0.58
AG6-10EEA	47,563,091	49.07	4,274	25,715	1465.94	373,768	94.10%	0.78
AG8-Rh89/T	56,054,626	48.56	11,793	31,591	3496.17	318,132	93.50%	0.53

Table S4. Taxonomic placement of *Rhizoctonia solani* and selected other basidiomycetes genomes used for comparative analysis					
**Sub-Division**	**Class**	**Subclass**	**Order**	**Family**	**Species, ENA Accession**
Agaricomycotina	Agaricomycetes		Cantharellales (*Incertae sedis*)	Ceratobasidiaceae	*Rhizoctonia solani* (Teleomorph: *Thanatephorus cucumeris*; soilborne plant pathogens)
				Tulasnellaceae	*Tullasnella calospora* (Patch-forming fungus; https://mycocosm.jgi.doe.gov/Tulca1/Tulca1.home .html)
			Polyporales (*Incertae sedis*)	Fomitopsidaceae	*Postia placenta* (Brown rot fungus; GCA_002117355)
				Ganodermaceae	*Ganoderma sinense* (Black Reishi; GCA_002760635)
			Agaricales	Agaricaeae	*Agaricus bisporus* (Button mushroom; GCA_000300555)
				Pleuroptaceae	*Pleurotus ostreatus* (Oyster mushroom; GCA_000697685)
				Amanitaceae	*Amanita muscaria* (Fly agaric; GCA_000827485)
				Omphalotaceae	*Lentinula edodes* (Shiitake; GCA_002003045)
					Hypsizygus marmoreus (Synonym: *H. tessellatus*; White clamshell mushroom; GCA_001605315)
				Physalacreaceae	*Armillaria solidipes* (Synonym: *A. ostoyae*; Humongous fungus; Wood or root decays of conifers; GCA_002307675)
	Tremellomycetes		Tremellales	Tremellaceae	*Cryptococcus neoformans* (Teleomorph: *Filobasidiella neoformans*; Animal pathogen; GCA_002215885)
	Wallemiomycetes (*incertae sedis*)				*Wallemia ichthyophaga (Halophilic fungus;* GCA_000400465*)*
Pucciniomycotina	Pucciniomycetes		Pucciniales	Pucciniaceae	*Puccinia graminis* (Stem rust; GCA_000149925.1)
				Melampsoraceae	*Melampsora larici-populina* (Poplar leaf rust; GCA_000204055.1)
Ustilaginomycotina	Exobasidiomycetes	Exobasidi omycetida e	Tilletiales	Tilletiaceae	*Tilletia caries* (Common bunt; GCA_001645005)
	Ustilaginomycetes		Ustilaginales	Ustilaginaceae	*Ustilago maydis* (Corn smut; GCA_000328475.2)

Table S5: Summary of ENA accession IDs assigned to individual genome assemblies.			
Assembly Name	ENA Accession	ENA Study ID	ENA Sample ID
AG1-1A	GCA_905219565	PRJEB42614	ERS5595528
AG1-1B	GCA_905219545	PRJEB42614	ERS5595527
AG1-1C	GCA_905219585	PRJEB42614	ERS5595529
AG2-2IIIB	GCA_905219575	PRJEB42614	ERS5595530
AG3-1A1	GCA_905219595	PRJEB42614	ERS5595531
AG3-1AP	GCA_905219605	PRJEB42614	ERS5595537
AG3-T5	GCA_905219615	PRJEB42614	ERS5595532
AG4-R118	GCA_905219625	PRJEB42614	ERS5595533
AG4-RS23	GCA_905219635	PRJEB42614	ERS5595534
AG5	GCA_905232135	PRJEB42614	ERS5595535
AG6-10EEA	GCA_905219655	PRJEB42614	ERS5595536
AG8	GCA_905219645	PRJEB42614	ERS5595538

References

1. Amaradasa,B.S., Lakshman,D., Horvath,B.J. and Amundsen,K.L. (2014) Development of SCAR markers and UP-PCR cross-hybridization method for specific detection of four major subgroups of Rhizoctonia from infected turfgrasses. *Mycologia*, 10.3852/13-006.
2. Lakshman,D.K. and Tavantzis,S.M. (1994) Spontaneous appearance of genetically distinct double-stranded RNA elements in Rhizoctonia solani. *Phytopathology*, 10.1094/Phyto-84-633.
3. Cubeta,M.A., Thomas,E., Dean,R.A., Jabaji,S., Neate,S.M., Tavantzis,S., Toda,T., Vilgalys,R., Bharathan,N., Fedorova-Abrams,N., *et al.* (2014) Draft genome sequence of the plant-pathogenic soil fungus Rhizoctonia solani anastomosis group 3 strain Rhs1AP. *Genome Announc.*, 10.1128/genomeA.01072-14.
4. Johnk,J.S., Jones,R.K., Shew,H.D. and Carling,D.E. (1993) Characterization of populations of Rhizoctonia solani AG-3 from potato and tobacco. *Phytopathology*, **83**, 854–858.
5. Papavizas, G. C. and Ayers,W.A. (1965) Virulence, host range, and pectolytic enzymes of single-basidiospore isolates of Rhizoctonia praticola and Rhizoctonia solani. *Phytopathology*, **55**, 111–116.
6. Lewis,J.A. and Papavizas,G.C. (1987) Reduction of inoculum of Rhizoctonia solani in soil by germlings of Trichoderma hamatum. *Soil Biol. Biochem.*, 10.1016/0038-0717(87)90081-2.
7. Lakshman,D.K., Roberts,D.P., Garrett,W.M., Natarajan,S.S., Darwish,O., Alkharouf,N., Pain,A., Khan,F., Jambhulkar,P.P. and Mitra,A. (2016) Proteomic Investigation of Rhizoctonia solani AG 4 Identifies Secretome and Mycelial Proteins with Roles in Plant Cell Wall Degradation and Virulence. *J. Agric. Food Chem.*, 10.1021/acs.jafc.5b05735.

Supplementary figures

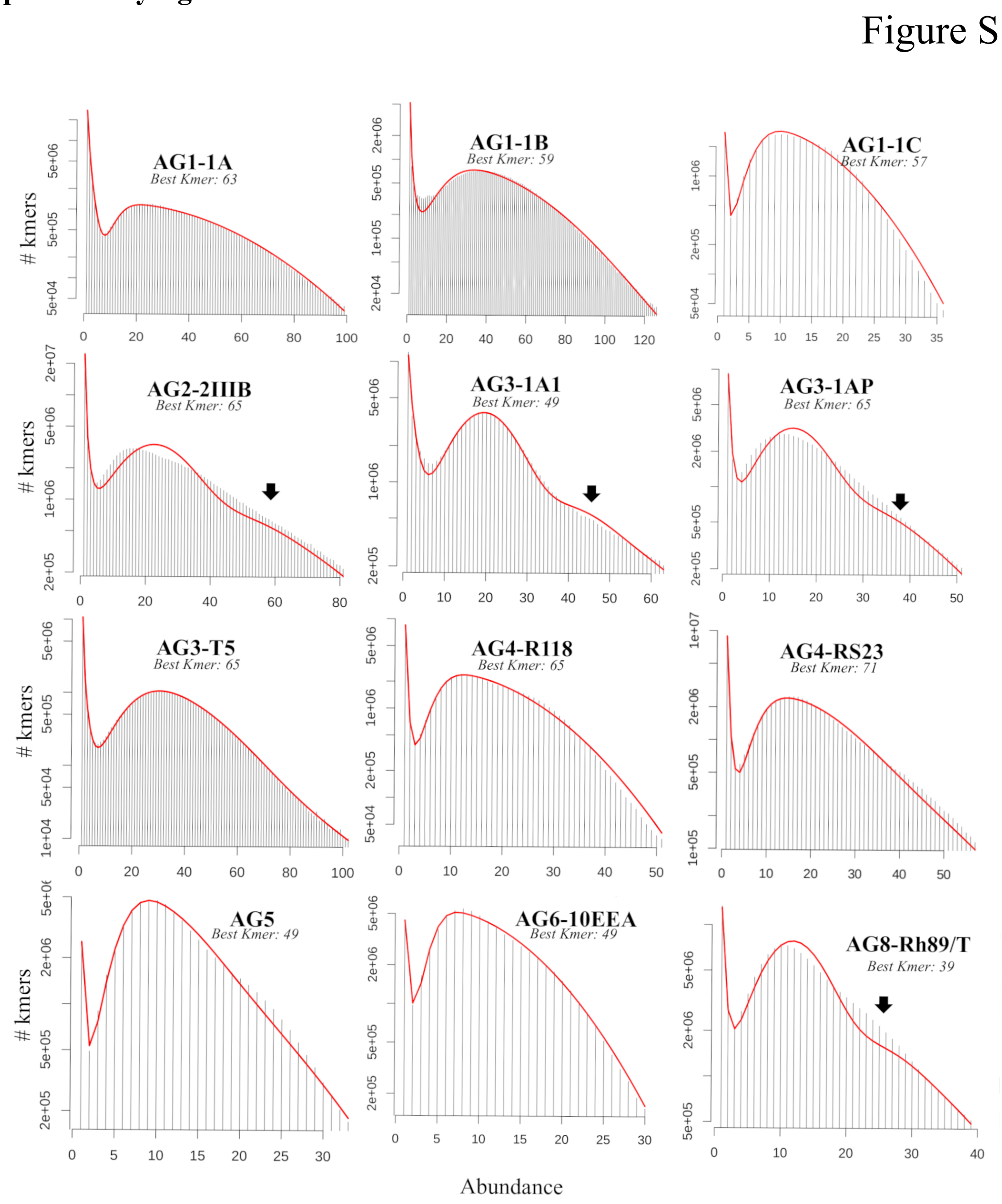

Figure S1. *k*-mer distribution. Histogram with the number of k-mers having a given count for the best *k*-mer length, plotted using –histo module of jellyfish program. The x-axis represent number of *k*-mers observed with a given *k*-mer count (y-axis). Arrow marks the shoulder peaks that represent sequence-level heterogeneity or multi-nucleate features in the genome sequencing data.

Figure S2

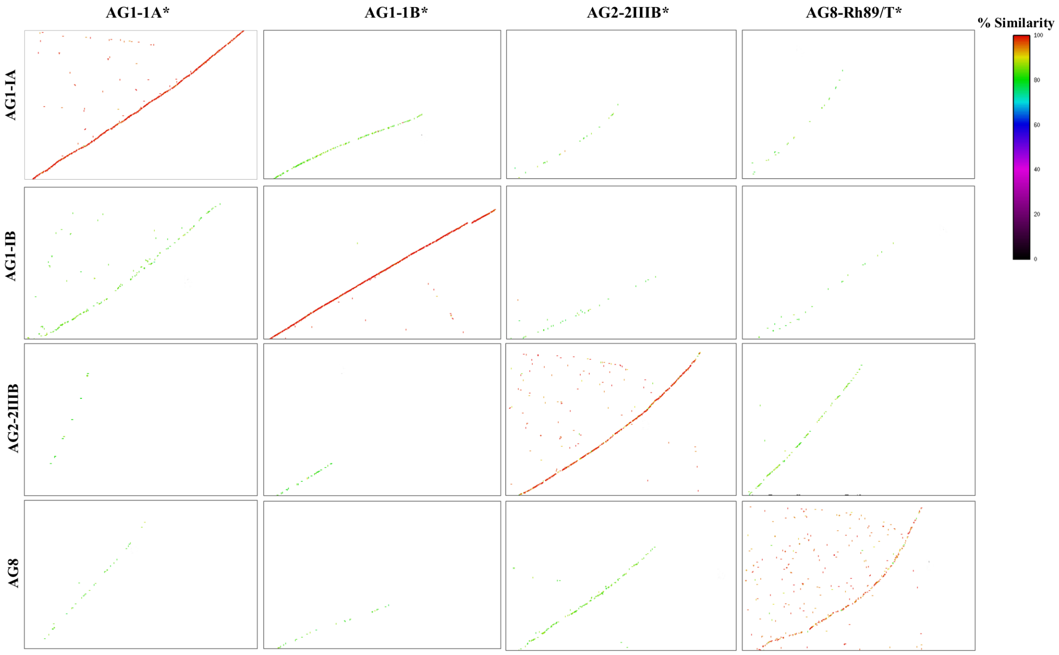

Figure S2: Mummer plots of aligned genomic scaffolds of *R. solani* AGs between previously published genomes and de novo genome assemblies (marked with *) produced in this study. The red dots represent high sequence similarity between aligned genomic scaffolds. The scaffolds were aligned using nucmer program and plotted with mummer plot with –filter and –layout arguments.

Figure S3

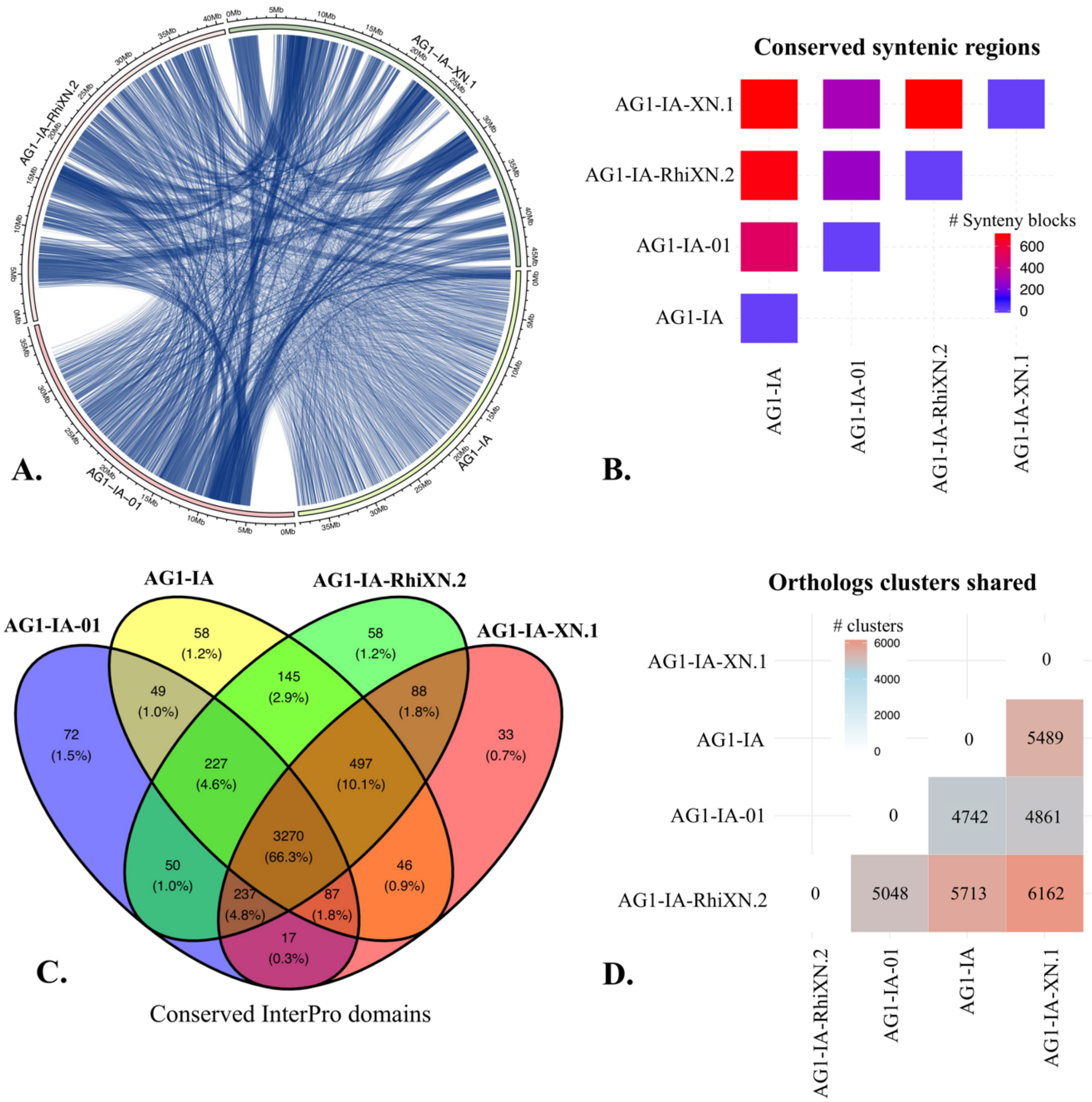

Figure S3. **AG1 subgroup comparison**. **A.** The Circos plot represents the syntenic relationship between genomes of different AG1 subgroups of *Rhizoctonia solani* Kühn. Each line represents the region of genomic similarity predicted with Synima (Method: RHB). Only the regions with coverage > 40,000 bases were enumerated and shown. **B.** The heatmap highlights the number of high-similarity syntenic regions (coverage > 40,000 bp) shared between each pair of AG1 genomes. AG1-1A shares a large number of syntenic relationships to other pairs of isolates, despite having sparse scaffolds than other assemblies. Here, self-hits were removed or not shown. **C.** Comparative analysis of InterPro domains conserved in the AG1 subgroup assemblies. AG1-1A shares ~70% or more of InterPro domains with at least one other AG1 subgroup assembly, under comparison. **D.** The number of protein ortholog clusters shared between any two isolates. A shared cluster means, a given cluster contains proteins from a given pair of isolates.

Figure S4

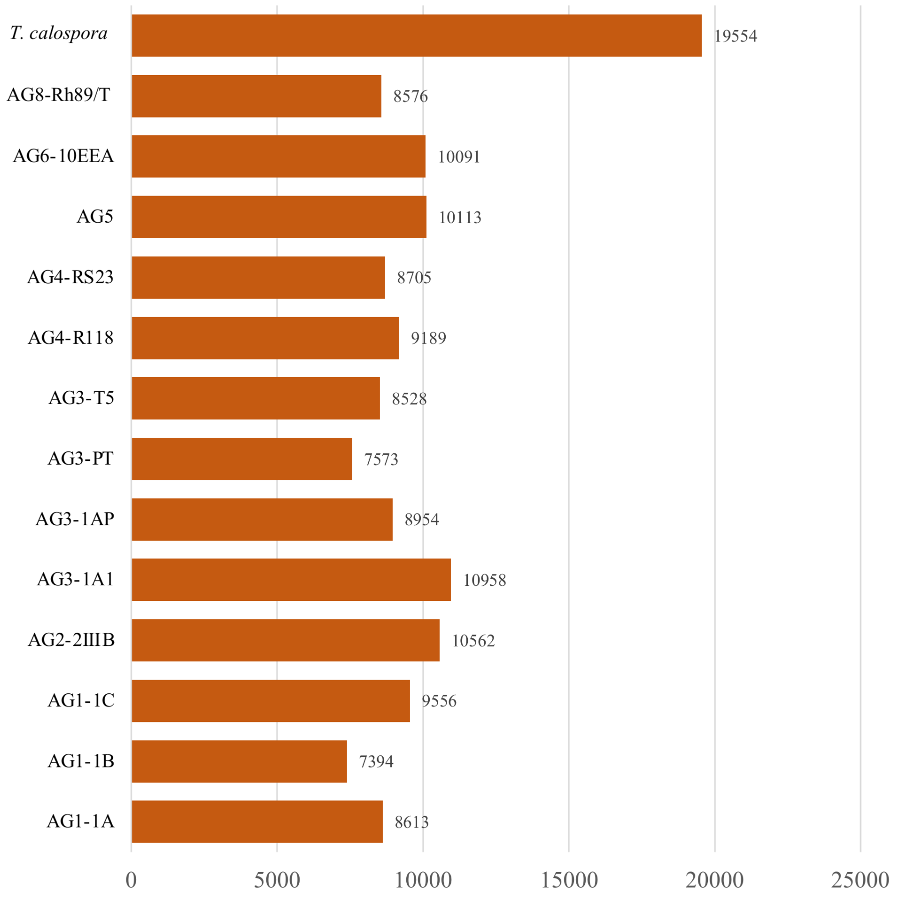

Figure S4. The number of protein coding transcripts/proteins predicted in each of the given isolate used in this study.

Figure S5

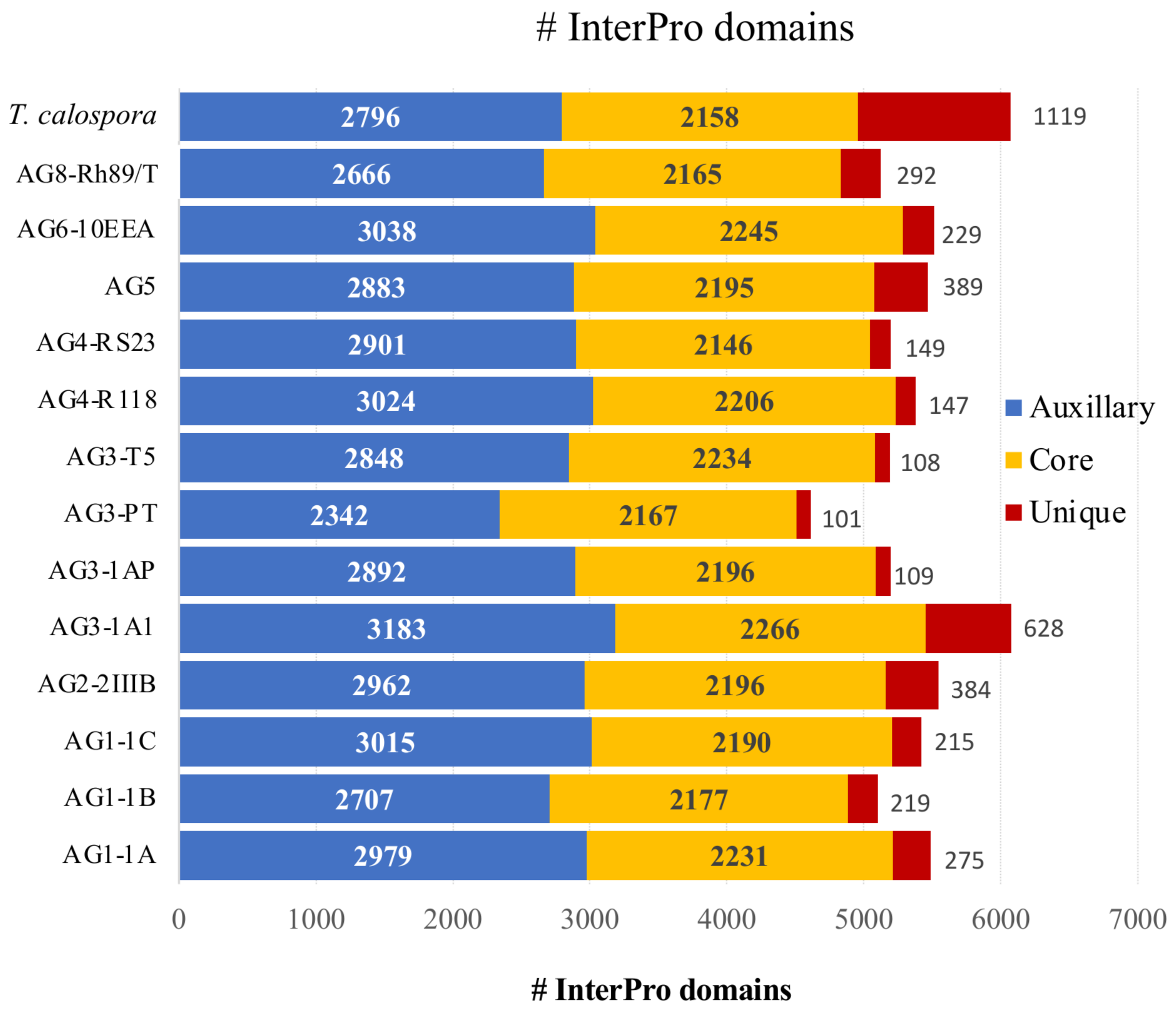

Figure S5: **Interpro domain families**. In each isolate, for each type of protein (core, unique and auxiliary), interpro domain families were enumerated. The stacked bar plot shows the number of interpro families enriched with the different protein types in each fungal isolate.

Figure S6

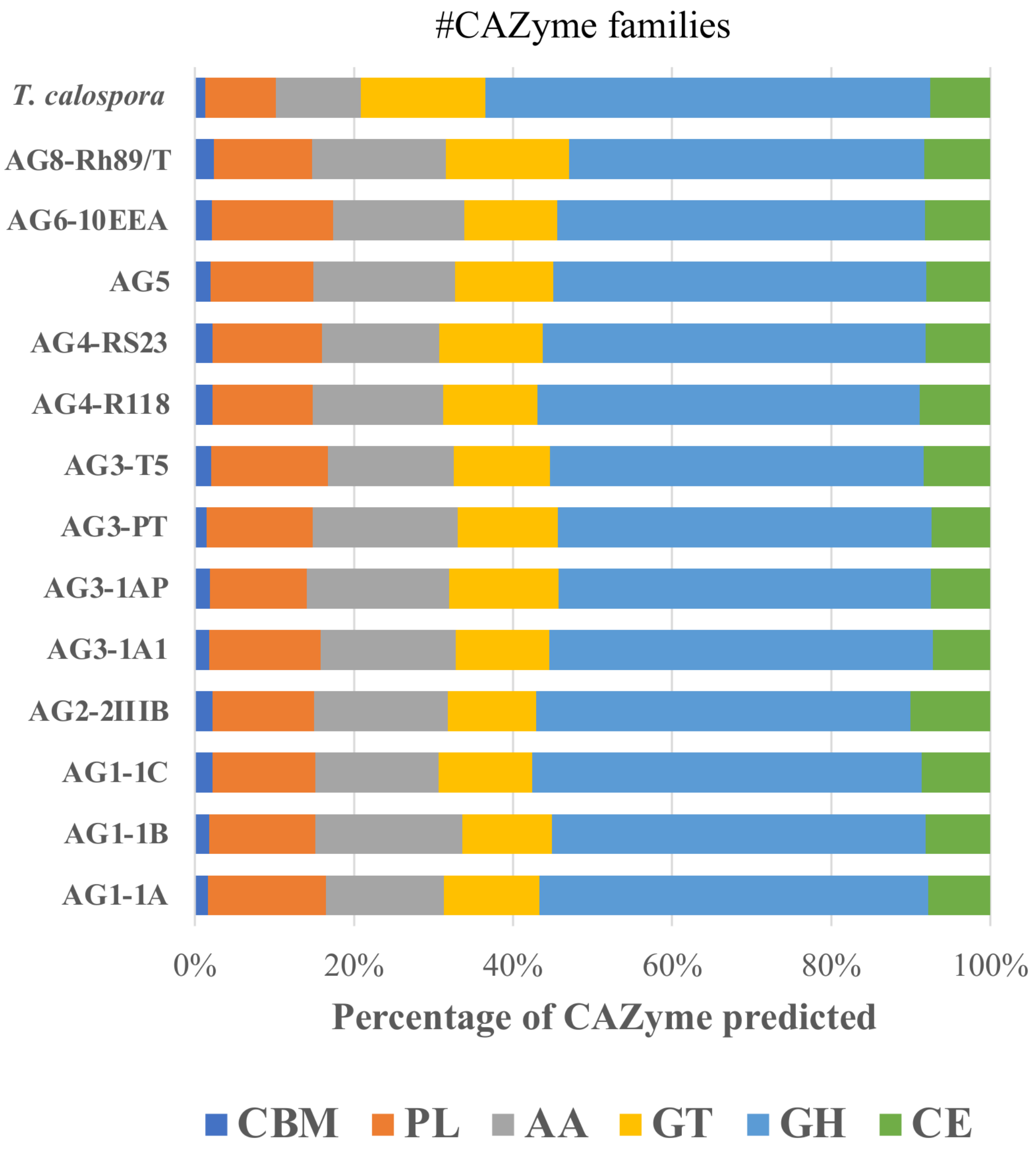

Figure S6: CAZyme families. The proportion of proteins, belonging to six different carbohydrate metabolizing families of proteins, predicted in each of the given fungal isolates.

Figure S7

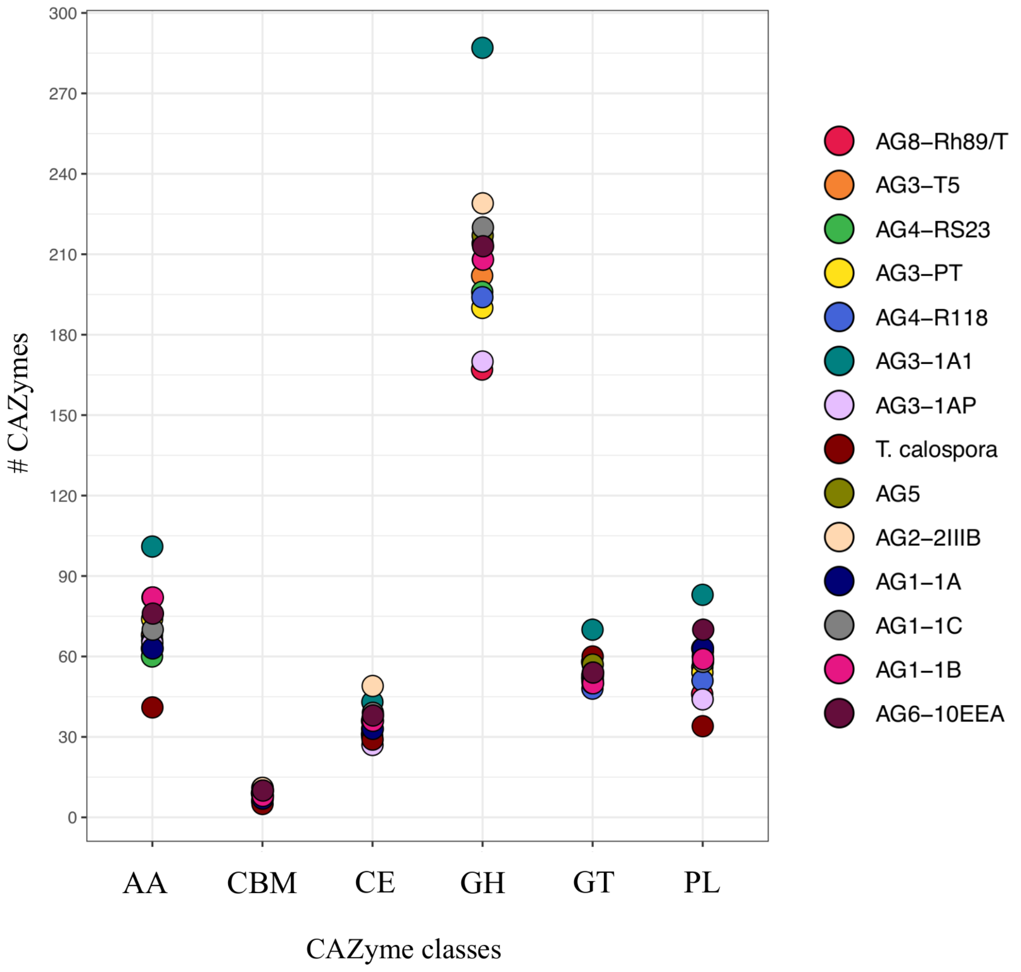

Figure S7. CAZyme. The number of CAZymes belonging to different classes, predicted in *R. solani* isolates *and T calospora.*

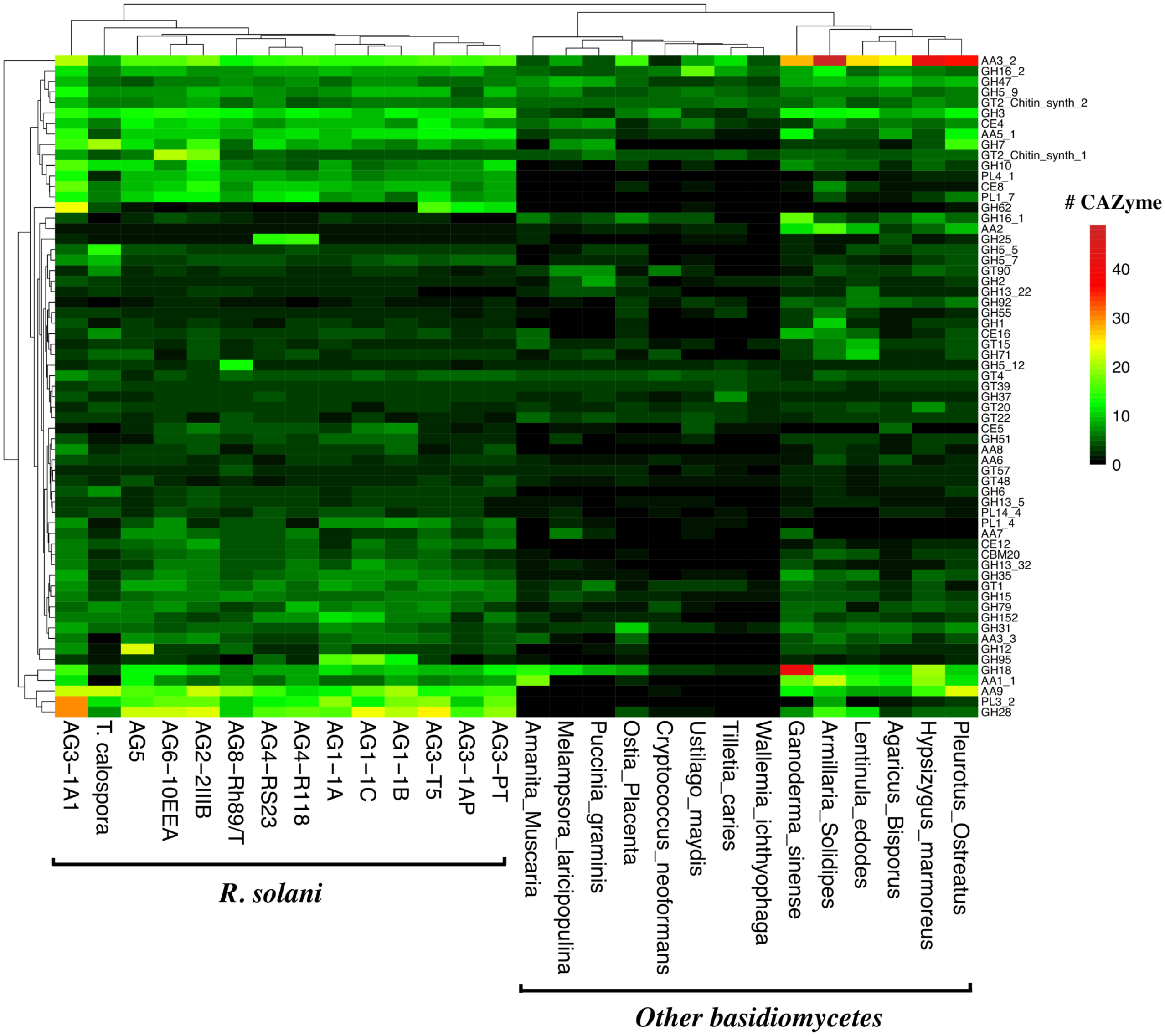

Figure S8. **CAZyme**. Heatmap showing the topmost CAZyme family predicted in the proteomes of all the *R solani* AGs, *T calospora* and other basidiomycetes. Each row represents one CAZy family of proteins, and color is proportional to the number of protein member shared in a given family from the given species (black: no member protein; red: large number of members). The phylogenetic analysis enumerates the distance between different fungal isolates based on proteins shared by them across all CAZy families. For simplicity only the CAZyme families enriched in more than 50 proteins across all proteomes were shown.
